# One-stage bilateral implantation of a calcar-guided short-stem in total hip arthroplasty: Minimally invasive modified anterolateral approach in supine position

**DOI:** 10.1007/s00064-016-0481-5

**Published:** 2017-02-03

**Authors:** K. P. Kutzner, S. Donner, M. Schneider, J. Pfeil, P. Rehbein

**Affiliations:** Department of Orthopaedic Surgery and Traumatology, St. Josefs Hospital, Beethovenstraße 20, 65189 Wiesbaden, Germany

**Keywords:** Arthroplasty, replacement, hip, Prostheses and implants, Minimally invasive surgical procedures, Round-the-corner, Optimys, Hüfttotalendoprothese, Prothesen und Implantate, Minimal-invasive Operationsverfahren, Round-the-corner, Optimys

## Abstract

**Objective:**

One-stage bilateral, muscle-preserving, calcar-guided implantation technique through the modified minimally invasive anterolateral approach in supine position.

**Indications:**

Bilateral primary/secondary osteoarthritis of the hip; bilateral femoral head necrosis; ASA I–III.

**Contraindications:**

ASA IV; severe osteoporosis, other factors jeopardizing stable anchorage of cementless, calcar-guided short-stem; infection.

**Surgical technique:**

Supine position. Skin incision. Opening of fascia; blunt dissection, pushing gluteal muscles dorsally with the index finger. Capsulectomy. Individual osteotomy according to preoperative plan to determine short-stem position. Remove femoral head. Prepare acetabulum. Position cup. Femoral preparation with the curved opening awl. Spare greater trochanter and gluteal muscles. Insert trial rasps in ascending sizes with “round-the-corner” technique. Select offset version, then trial reposition with intraoperative radiograph and implantation of the definitive implant. Wound closure. Consultation with the anesthesiologist to confirm a stable patient. Same procedure on contralateral hip.

**Postoperative management:**

Mobilization on day 1 with immediate full weight bearing. Remove wound drains and urinary catheter (only female patients) on day 2. Intensive protocol of physiotherapy and rehabilitation. Thrombosis prophylaxis. Rehabilitation from day 7.

**Results:**

Almost 500 patients have undergone surgery since 2010. First consecutive 54 patients (108 hips) prospectively evaluated. After 2 years, Harris Hip Score was 98.8; satisfaction on visual analogue scale was 9.9. Low peri- and postoperative complication rates; no implant revisions.

**Conclusion:**

The muscle-sparing approach and the special “round-the-corner” technique in one-stage bilateral procedure leads to rapid mobilization and rehabilitation with excellent early clinical results and high satisfaction rates.

## Zusammenfassung

**Operationsziel:**

Die simultan-beidseitige, muskelschonende, kalkargeführte Implantation eines Kurzschafts über einen modifizierten, minimal-invasiven anterolateralen Zugang in Rückenlage.

**Indikationen:**

Bilaterale primäre und sekundäre Coxarthrose; bilaterale Hüftkopfnekrose; ASA I–III.

**Kontraindikationen:**

ASA IV; deutliche Osteoporose bzw. andere Faktoren, welche eine sichere Verankerung eines zementfreien Kurzschafts gefährden; Infektion.

**Operationstechnik:**

Rückenlagerung. Hautschnitt. Längsinzision der Faszie, stumpfe Präparation der Glutealmuskulatur nach dorsal mit dem Zeigefinger. Entfernen der ventralen Kapsel, individuelle Osteotomie entsprechend der präoperativen Planung, um gewünschte Schaftpositionierung zu erreichen. Extraktion des Hüftkopfes, Präparation des Azetabulums. Pfannenpositionierung. Femorale Präparation mittels abgerundeter Eröffnungsahle, vollständige Schonung des Trochanter major sowie der Glutealmuskulatur. Aufraspeln in der „Round-the-corner“-Technik. Auswahl der gewünschten Offset-Version, Probereposition und intraoperative Röntgenkontrolle. Implantation der Originalimplantate und finale Reposition. Nach Wundverschluss, Rücksprache mit dem Anästhesisten. Wiederholen der gleichen Schritte auf der Gegenseite.

**Postoperative Behandlung:**

Mobilisation unter schmerzadaptierter Vollbelastung an Tag 1. Drainagezug und Entfernung des Blasenkatheters (nur für weibliche Patienten) spätestens an Tag 2. Intensives, standardisiertes physiotherapeutisches Protokoll. Leitliniengerechte Thromboseprophylaxe. Entlassung in stationäre oder ambulante Rehabilitation ab Tag 7.

**Ergebnisse:**

Der vorgestellte Kurzschaft wurde seit 2010 in nahezu 500 Fällen simultan-beidseits implantiert. Die ersten 54 konsekutiven Patienten (108 Hüften) wurden prospektiv in eine Studie eingeschlossen. Nach 2 Jahren betrug der Harris Hip-Score 98,8 und die Zufriedenheit auf der visuellen Analogskala 9,9. Sehr geringe peri- und postoperative Komplikationsrate. Bisher keine Implantatrevision.

**Schlussfolgerung:**

Die muskel- und weichteilschondende Kombination aus minimal-invasivem Zugang und spezieller „Round-the-corner“-Technik im Rahmen der simultan-bilateralen Implantation eines kalkargeführten Kurzschafts führt zu exzellenten klinischen Kurzzeitergebnissen und hoher Patientenzufriedenheit.

## Introduction

A recent analysis of the Swedish hip arthroplasty registry revealed that 17% of all patients receiving primary total hip arthroplasty (THA) suffer from bilateral symptoms of osteoarthritis [6]. One-stage bilateral THA is an alternative to staged unilateral THA in those patients; however, there is still broad concern about the safety and reliability of this procedure.

Given the presence of bilateral hip osteoarthritis accompanied with bilateral corresponding symptoms, one-stage bilateral THA offers various advantages for the patients [17]. Besides the necessity of only one surgical procedure and only one anesthesia, postoperative rehabilitation can be improved [9]. Bilateral treatment, in contrast to a staged unilateral procedure, leads to early painless ambulation, without any residual symptoms of the contralateral pathological hip. The quality of rehabilitation can be enhanced and duration in total can be reduced. In addition, the recent literature implicates a complication rate comparable or even lower than in the staged procedure [1, 19]. However, in order to ensure a safe procedure and high quality of postoperative function, one-stage bilateral THA needs to provide certain characteristics like short surgery duration, low blood loss and distinct muscle-sparing technique [14].

In modern THA raised consciousness in order to reduce muscle- and soft-tissue trauma has led to a widespread usage of minimally invasive approaches (MIS) [3, 8, 18]. Due to soft-tissue sparing techniques, some MIS approaches have been shown to possibly offer encouraging clinical results particularly in regard to early ambulation and blood loss [5, 22]. Besides smaller incisions, MIS techniques aim to reduce damage especially of the abductor muscles. The continuity consequently can be preserved. One of the most common approaches used in THA is the Watson–Jones anterolateral approach performed in the supine position [15]. Over time, modifications have led to the development of a MIS approach [4, 15]. It uses a muscular gap between tensor fasciae latae and gluteus medius without the necessity of any muscular transection. The gluteal muscles can be preserved. Consequently, it offers low blood loss, early recovery of hip function and excellent clinical short-term results [20, 21].

However, not only the type of approach ensures the muscular continuity and reduces damage to soft tissue, but also the type of implant. Modern calcar-guided short-stems have gained importance in recent years [7]. The development of new calcar-guided, metaphyseal anchoring short-stems amongst others pursues the strategy of being able to spare muscles, soft-tissue and bone [2]. They provide characteristics, making these implants well suitable for the usage in MIS techniques [15].

**Fig. 1:**
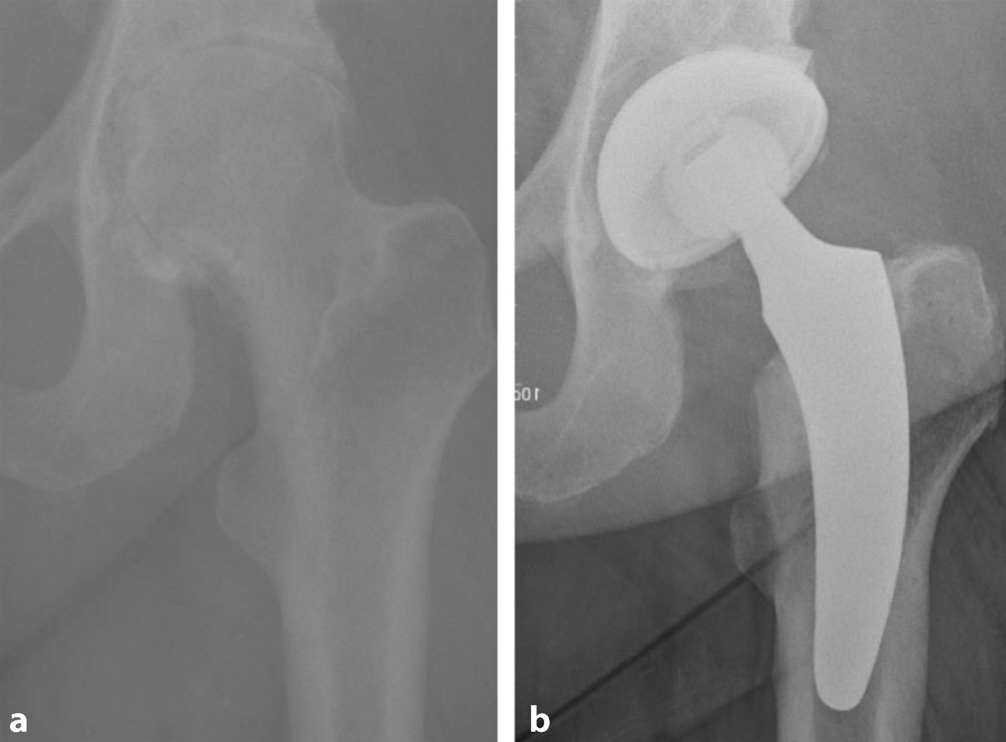
Valgus alignment. The short stem aligns itself according to the patient’s anatomy alongside the calcar in valgus position. Therefore most of the femoral neck is resected and the osteotomy is performed distally (**a** preop; **b** postop)

**Fig. 2:**
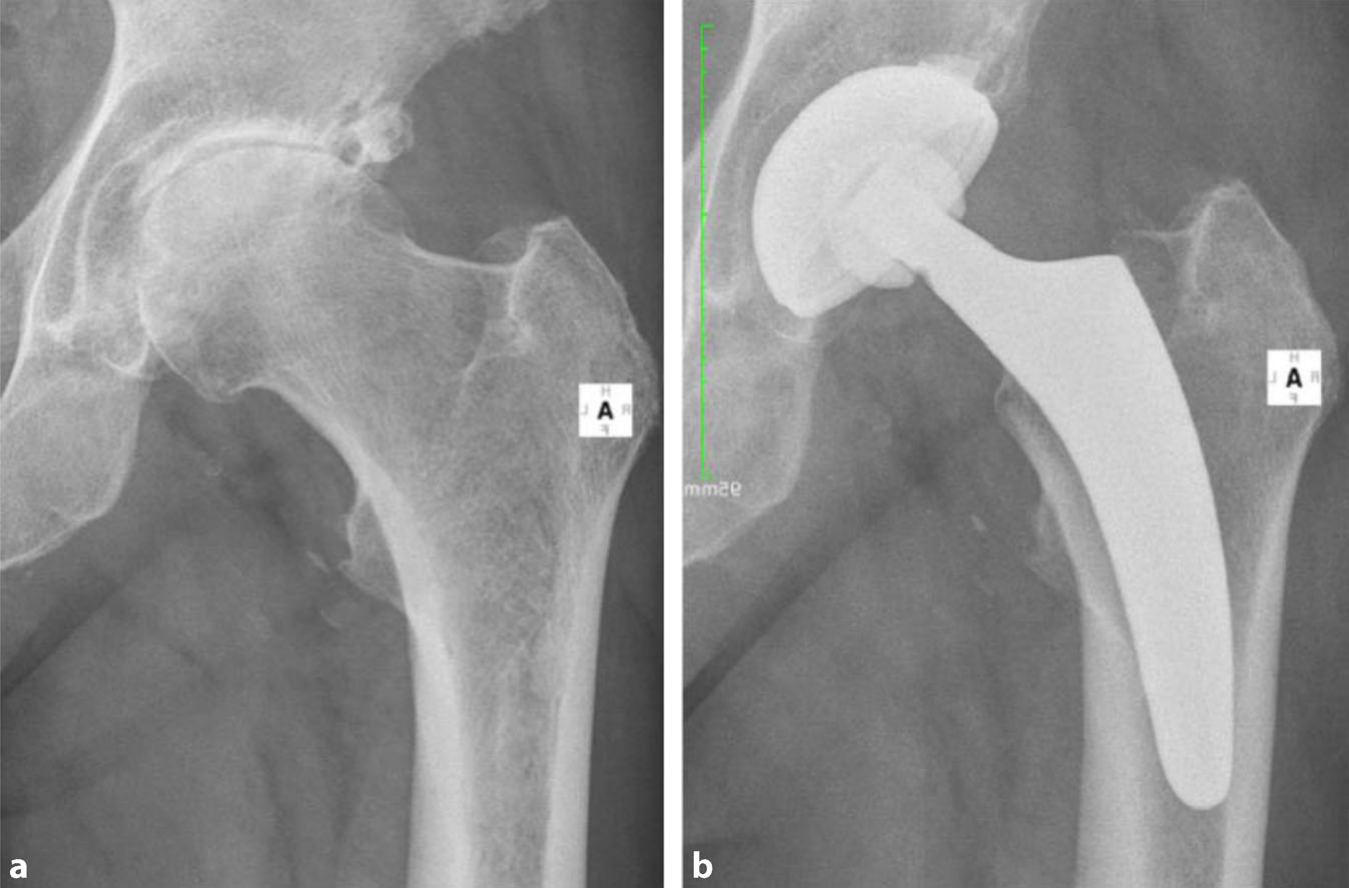
Varus alignment. The short stem aligns itself according to the patient’s anatomy alongside the calcar in varus position. Therefore the osteotomy is performed proximally partially preserving the femoral neck (**a** preop; **b** postop)

The key to these characteristics is a certain implantation technique which differs from conventional techniques used with traditional straight-stem designs. The heart of these implants, besides their reduced length, consists of the anatomical curvature, which has been adapted from the calcar. The positioning of the stem follows the personal anatomy alongside the calcar curve, making possible an individualized implantation ([10], Figs. 1 and 2). Employing a particular “round-the-corner” technique, the greater trochanter especially, together with the gluteal muscles can be distinctly protected [7].

Consequently the combination of MIS techniques accompanied with the usage of a caclar-guided short stem possibly offers distinct qualities regarding early clinical results and may support encouraging early functional outcomes [16]. Possibly the operation time and blood loss may be reduced to a low level. Several short-term results of different types of implants support these anticipations in the early stages [2, 9, 12, 13].

All these features taken together might be helpful especially in the demanding perioperative management of one-stage bilateral THA.

We describe the one-stage bilateral procedure of the muscle-preserving, calcar-guided implantation technique using a calcar-guided short-stem through the modified MIS anterolateral approach in supine position.

## Surgical principle and objective

The main goals in modern THA especially in one-stage bilateral procedures today are the sparing of bone and soft tissue, a fast and reliable technique and excellent early clinical results with possible high postoperative activity levels. The combination of modern calcar-guided short-stems using a modified MIS anterolateral approach aims to meet these requirements. The special “round-the-corner” technique of implantation without damage to the greater trochanter and the gluteal muscles is key.

## Advantages

### One-stage bilateral procedure

- Only one procedure
- Only one anesthesia
- Only one hospital stay
- Only one rehabilitation
- Early painless ambulation (no left-over pathological hip)
- Cost- and time-saving

### MIS-modified anterolateral approach

- No muscular transection is needed
- Damage to periarticular soft-tissues can be minimized
- Small skin incision
- Ensures fast rehabilitation; fast-track concepts are possible
- Blood loss can be minimized

### Calcar-guided short stems

- Implantation technique is well suitable for MIS approaches
- Allow full weight-bearing immediately after surgery
- Given the “round-the-corner” technique, greater trochanter region can be spared completely, protecting particularly the gluteal muscles
- Fewer fractures of the greater trochanter [12]
- Damage to periarticular soft-tissues can be minimized
- After learning curve, implantation technique is fast and easy
- Individual positioning of the implant alongside the calcar curve in order to reconstruct patient’s personal anatomy [10]
- Metaphyseal anchorage allows physiological load distribution [11]

## Disadvantages

### One-stage bilateral procedure

- Prolonged length of surgery
- Increased blood loss compared to unilateral procedure
- Spinal anaesthesia not recommended, due to limited effect duration and a possible decrease in muscle relaxation

### MIS-modified anterolateral approach

- Orthopaedic surgeon should be experienced in THA using MIS techniques
- Operation technique might vary depending on surgeon’s experience

### Calcar-guided short stems

- Implantation technique includes a learning curve
- Long-term results are lacking

## Indications

Bilateral appearance of

- Primary osteoarthritis of the hip
- Secondary osteoarthritis of the hip
- Necrosis of the femoral head (as long as stable anchorage is not jeopardized)
- ASA I–III

## Contraindications

- ASA IV
- Neurological disorders
- Severe osteoporosis
- Lack of stability
- Joint infection, systemic infection
- Severe bone-loss or bone-defects
- Severe anatomical deformities or abnormalities
- Severe obesity. This can be considered as relative contraindication because of generally enhanced perioperative risks and complication rates

## Patient information

- General surgery related risk factors
- Hip arthroplasty related risk factors
  - Infection, dislocation, leg length discrepancy, wear, fracture, aseptic or septic loosening
- Possible revision surgery
- Intraoperative switch to unilateral procedure
- Intraoperative switch to cementless straight-stem or cemented THA
- Estimated hospital stay of 7 days (dry wound without seroma and the ability to walk stairs independently should be achieved)
- Full weight-bearing protocol, intensive physical therapy
- Medical prophylaxis of thrombosis, analgesia
- Clinical and radiological follow-up after 6 weeks

## Preoperative work-up

- Assessment of medical history and clinical examination
- Range of motion (ROM)
- Leg length discrepancy, muscular deficiency, limping
- Assessment of neurological and vascular status
- Functional scores, e. g. Harris Hip Score (HHS) or Western Ontario and McMaster Universities Arthritis Index (WOMAC)
- Radiographs: deep pelvis anteroposterior and axial view both hips
- Preoperative templating is mandatory

## Instruments and implants

- Standard instruments for THA
- Specific instruments and implants for calcar-guided short-stem implantation (optimys, Mathys Ltd. Bettlach, Switzerland)
  - Specially curved opening awl
  - Double offset minimally invasive rasp handle (left and right)
  - Implant-shaped rasp (sized exactly like implant), also serves as trial implant
  - Trial cone (two different offset versions: standard and lateral offset)
  - Special implant impactor

## Anesthesia and positioning

**Fig. 3:**
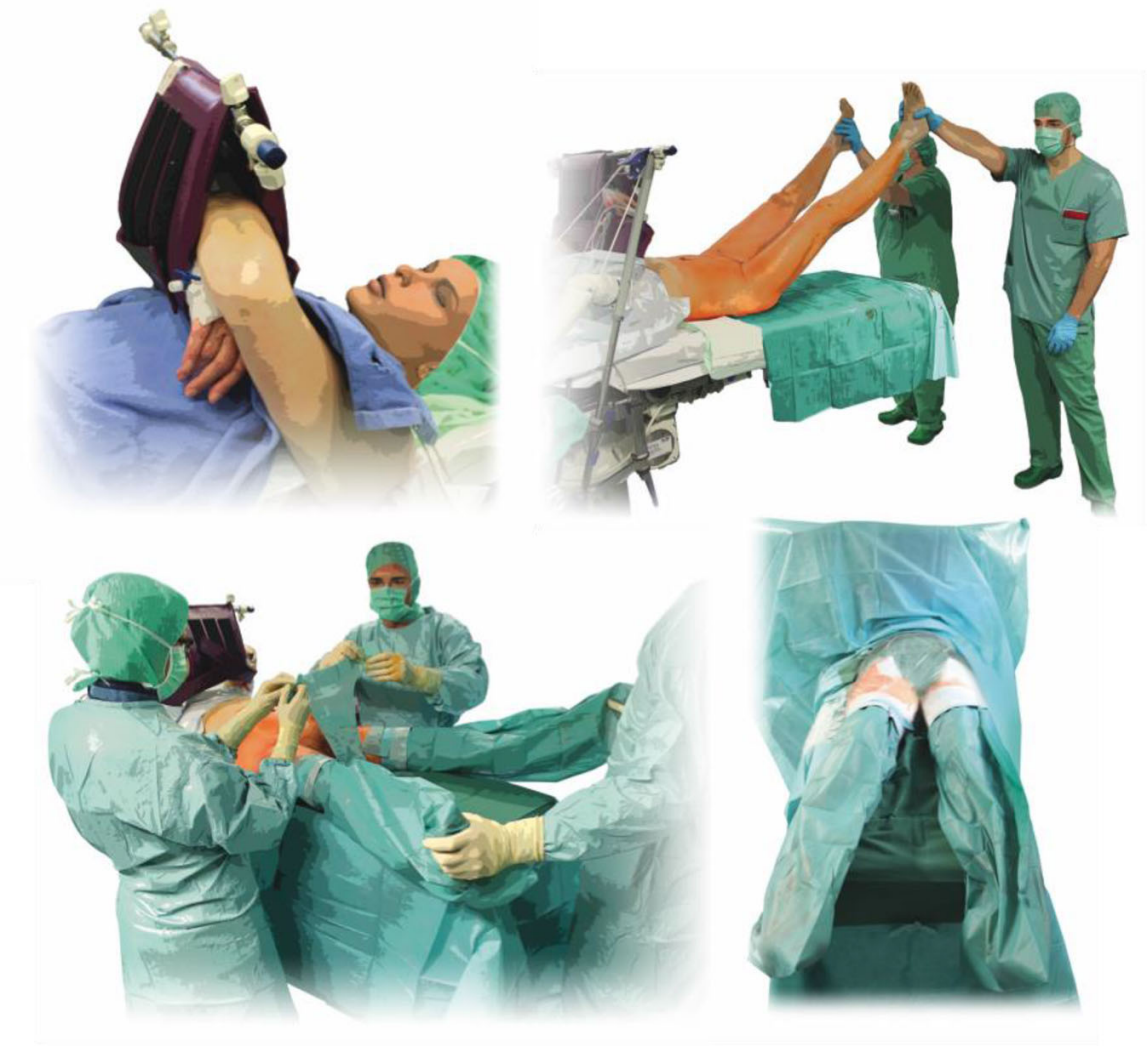
Patient positioning and sterile coverage: supine position on standard operating table with two separate leg supports. Legs remain mobile during surgery. (Courtesy of Mathys Ltd Bettlach)

**Fig. 4:**
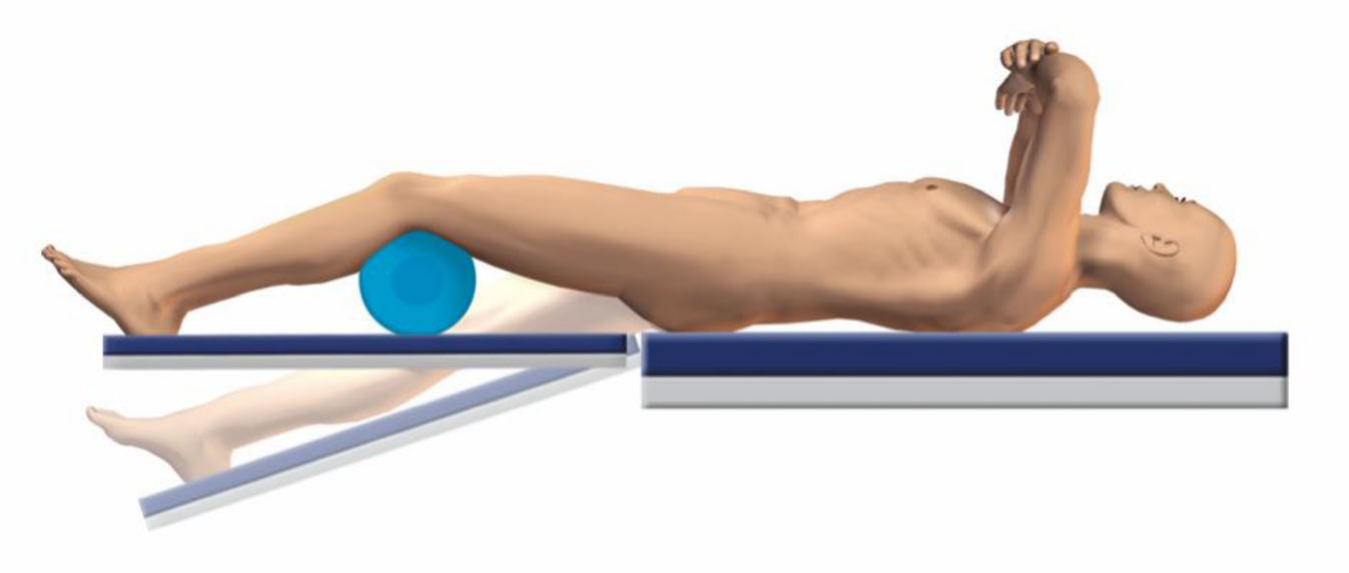
Starting with the first hip the ipsilateral side is slightly flexed using a knee roll. In addition for the femoral preparation the contralateral side is hyperextended (15°). (Courtesy of Mathys Ltd Bettlach)

- General anesthesia
- Spinal anesthesia not recommended in one-stage bilateral procedure due to limited effect duration
- Single-shot antibiotic therapy preoperatively (e. g. cephalosporin)
- Tranexamic acid (1 g i. v.) preoperatively, repetition if operation time exceeds 90 min (with regard to contraindications)
- Supine position; the patient is positioned on the operating table on the ipsilateral edge of the side to start with. After switching to contralateral side the patient is moved over to the contralateral edge respectively
- Standard operating table, two separate leg supports (Fig. 3)
- Slight abduction (10°) of the contralateral leg for acetabular preparation, hyperextension (15°) for femoral preparation
- During acetabular preparation a knee roll is placed below the ipsilateral knee (Fig. 4)
- Before starting with the second hip, the anesthesiologist is consulted in order to confirm a safe further procedure

## Surgical technique

(Figs. 5, 6, 7, 8, 9, 10, 11, 12, 13, 14, 15, 16, 17, 18, 19, 20, 21, 22, 23, 24).

**Fig. 5:**
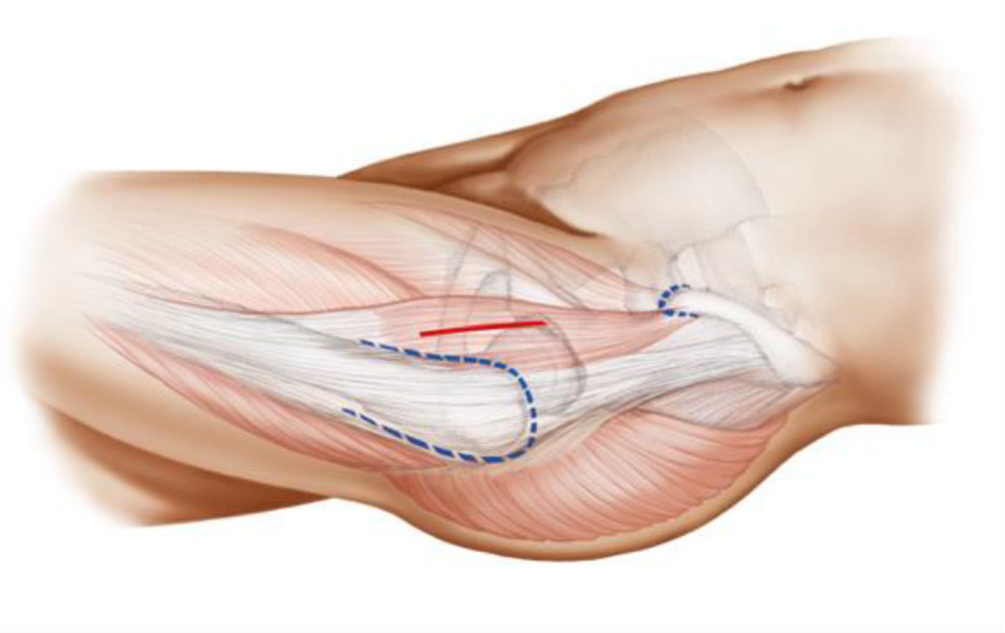
The anterior superior iliac spine is located and palpated. Skin incision (about 6 cm) is centered on the anterior tip of the greater trochanter aiming at the anterior superior iliac spine above the intermuscular septum between gluteus medius and the tensor fasciae latae. (Courtesy of Mathys Ltd Bettlach)

**Fig. 6:**
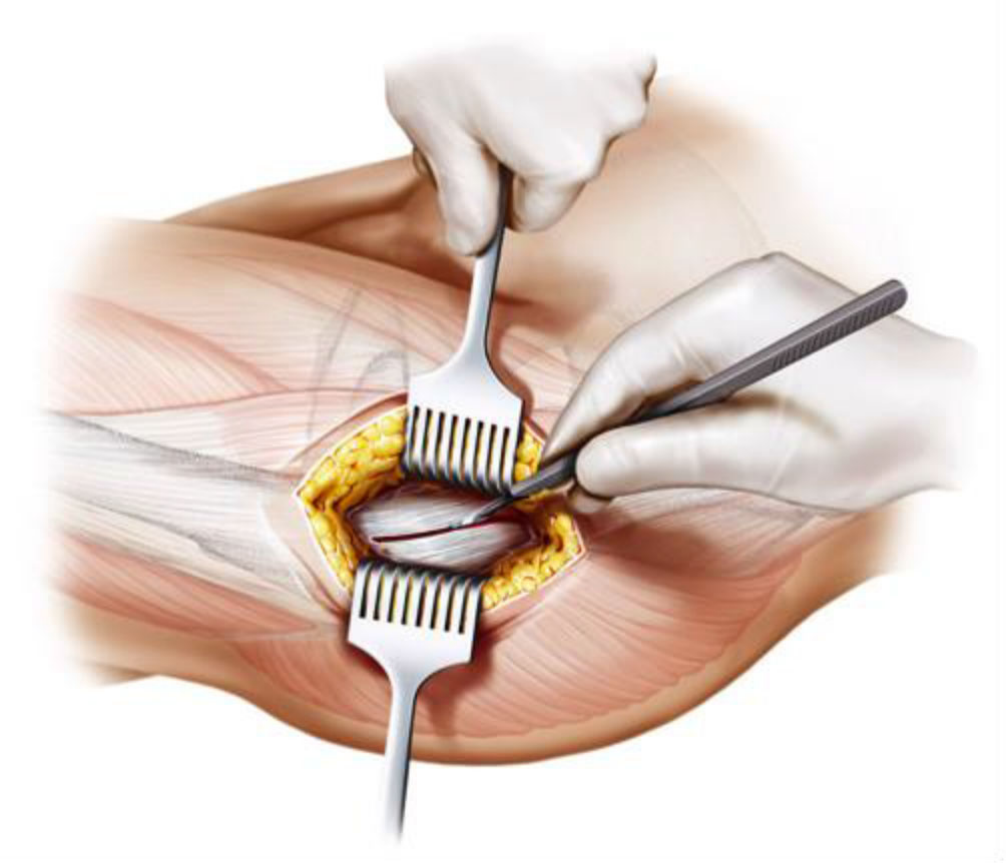
After incision of subcutaneous fat, two skin retractors are used. The fascia is opened without causing damage to the tensor fasciae latae. From [15]

**Fig. 7:**
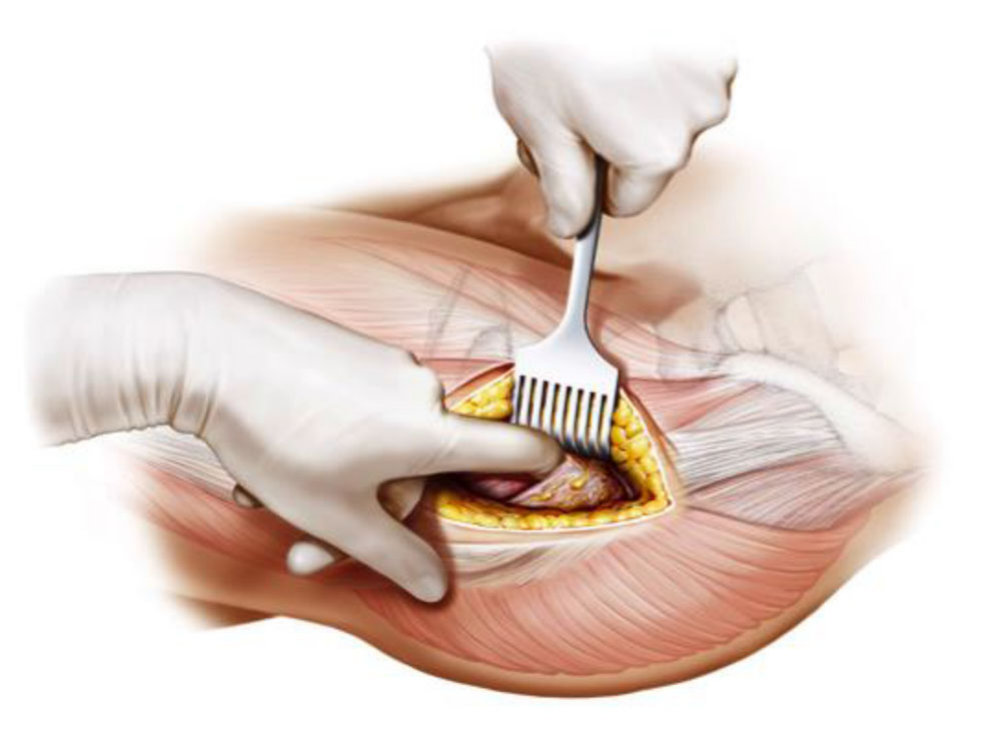
Using the index finger reaching the anterior upper part of the capsule a blunt dissection is done. Gluteal muscles are pushed posteriorly without damage. From [15]

**Fig. 8:**
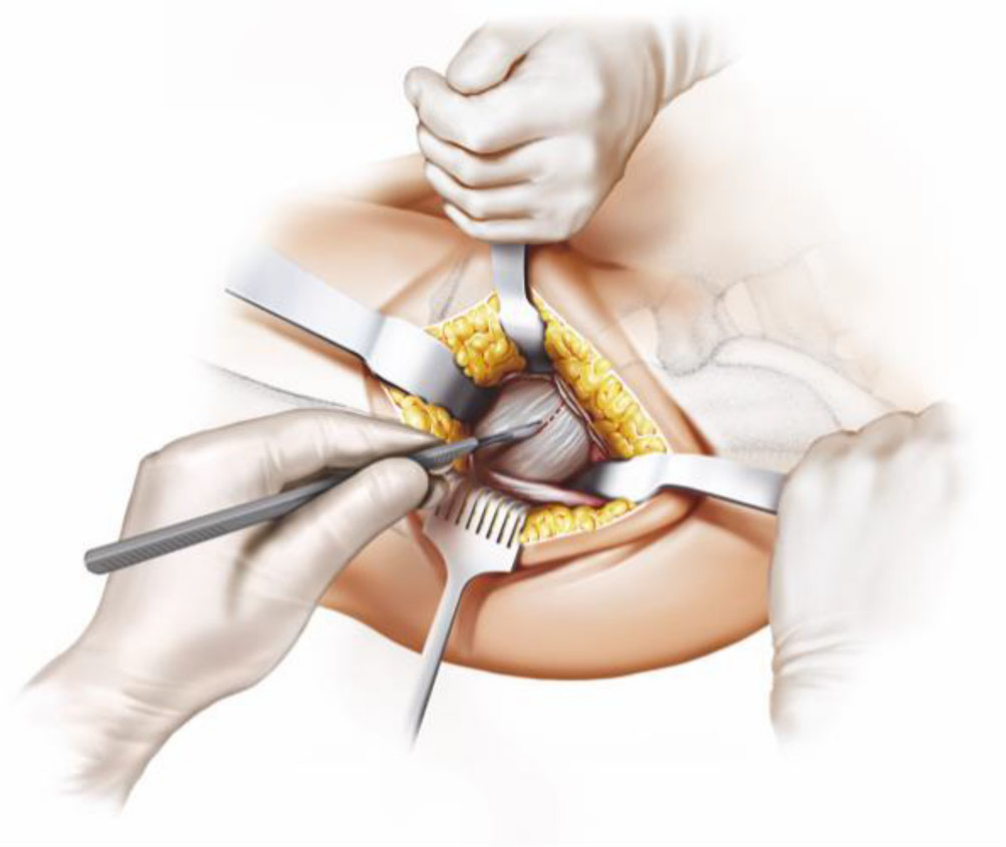
The joint capsule is exposed by two curved Hohmann retractors without sharp edges positioned cranial and caudal the capsule. In addition one Hohmann retractor is positioned at the anterior rim of the acetabulum, medializing vastus lateralis muscle without damage. The incision is done alongside of the femoral neck and the capsulectomy is performed. Note that no sharp dissection of any muscle, especially the gluteal muscles, is needed. From [15]

**Fig. 9:**
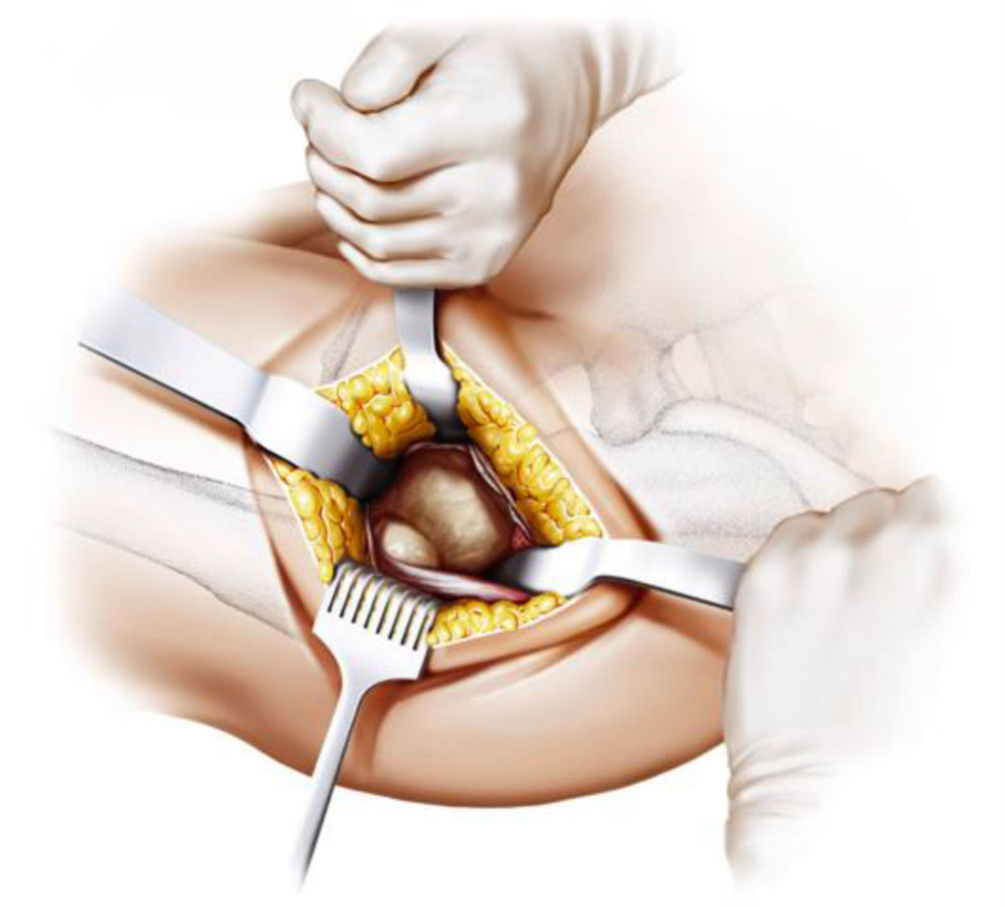
After removal of the anterior joint capsule, the femoral neck is exposed in order to perform the osteotomy by placing the two facing curved retractors intracapsular around the femoral neck. From [15]

**Fig. 10:**
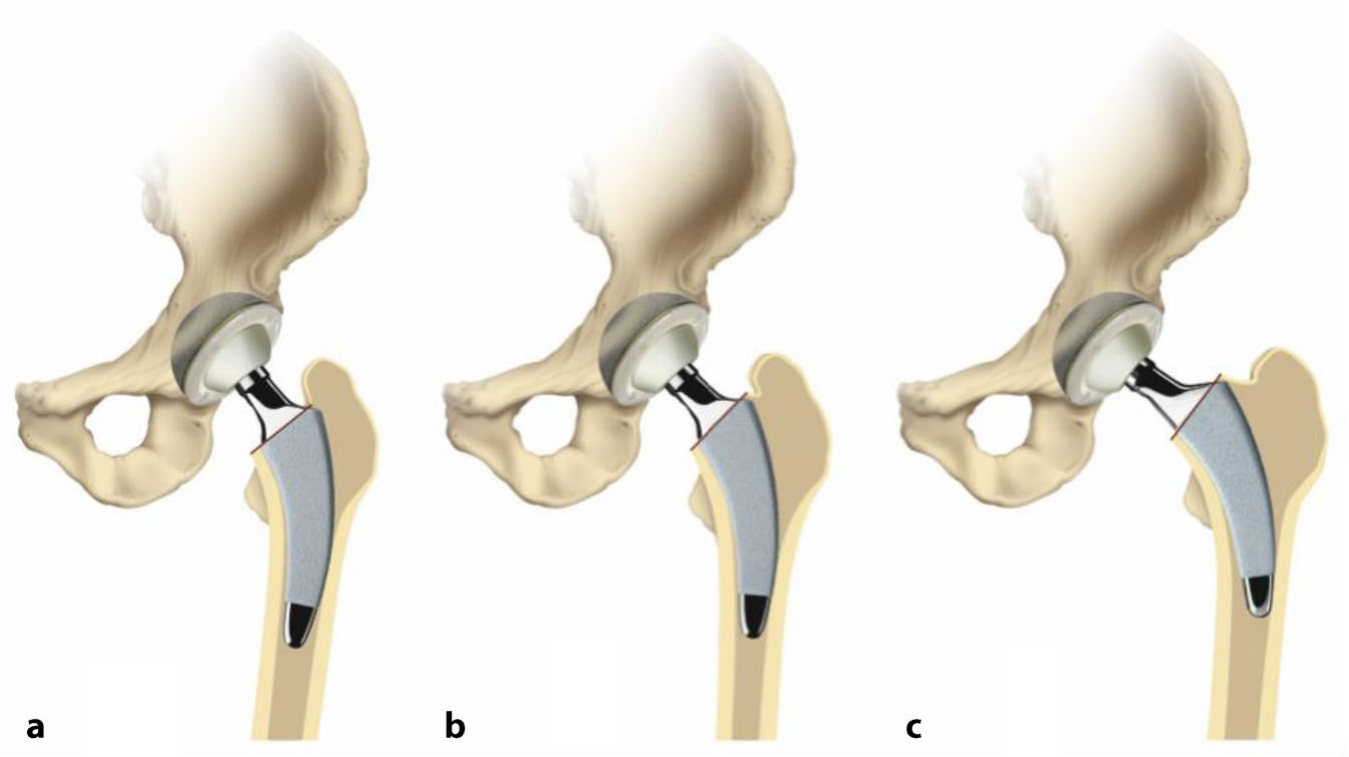
(**a** valgus**, b** neutral*,* **c** varus) One of the most important steps in implanting a calcar-guided short-stem is choosing the individual height of the osteotomy in order to partly preserve the femoral neck. Consequently a preoperative templating is mandatory. The height of the osteotomy is determined intraoperatively by palpation of the lesser trochanter and the fossa piriformis. In order to position the stem in a valgus position most of the femoral neck is resected and the osteotomy is performed distally (**a**). If the stem is to be implanted in a varus position the osteotomy is done proximally, according to the preoperative templating, preserving most of the femoral neck (**c**). This way femoral offset and leg length can be reconstructed in a large bandwidth [10]. From [15]

**Fig. 11:**
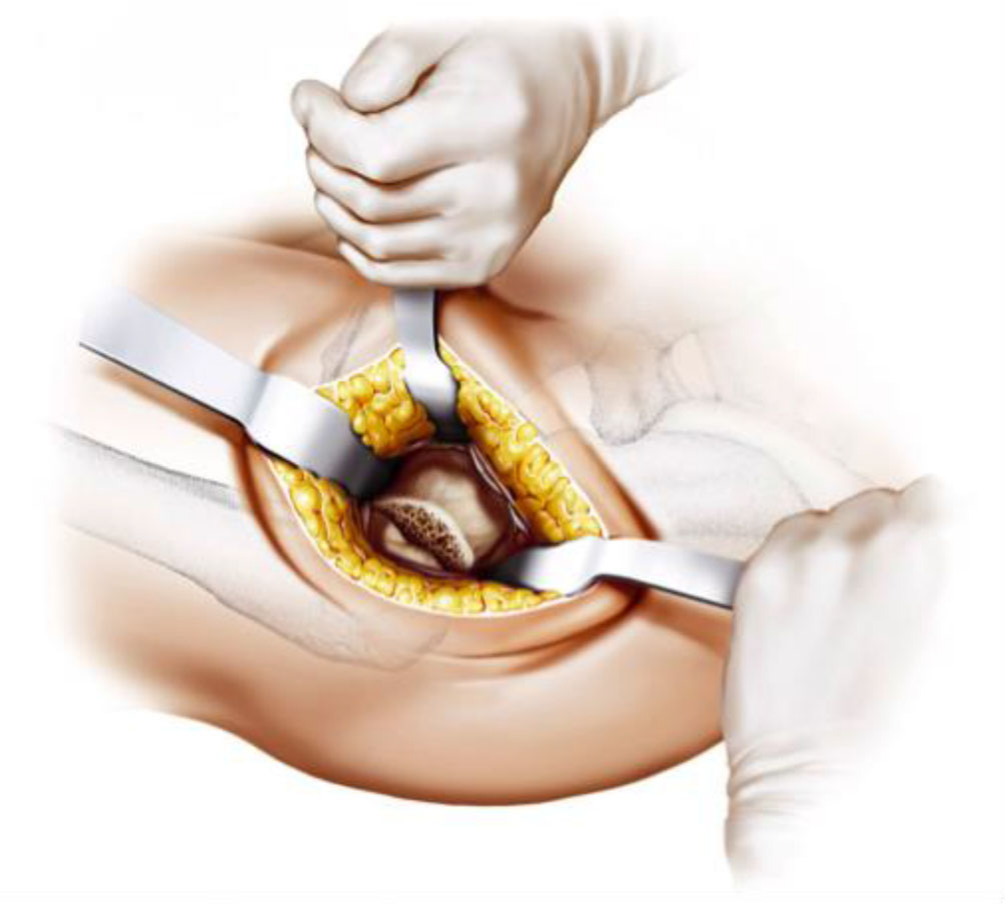
The osteotomy is done in slight external rotation of the ipsilateral leg according to the preoperative templating using a long stiff bladed oscillating saw. From [15]

**Fig. 12:**
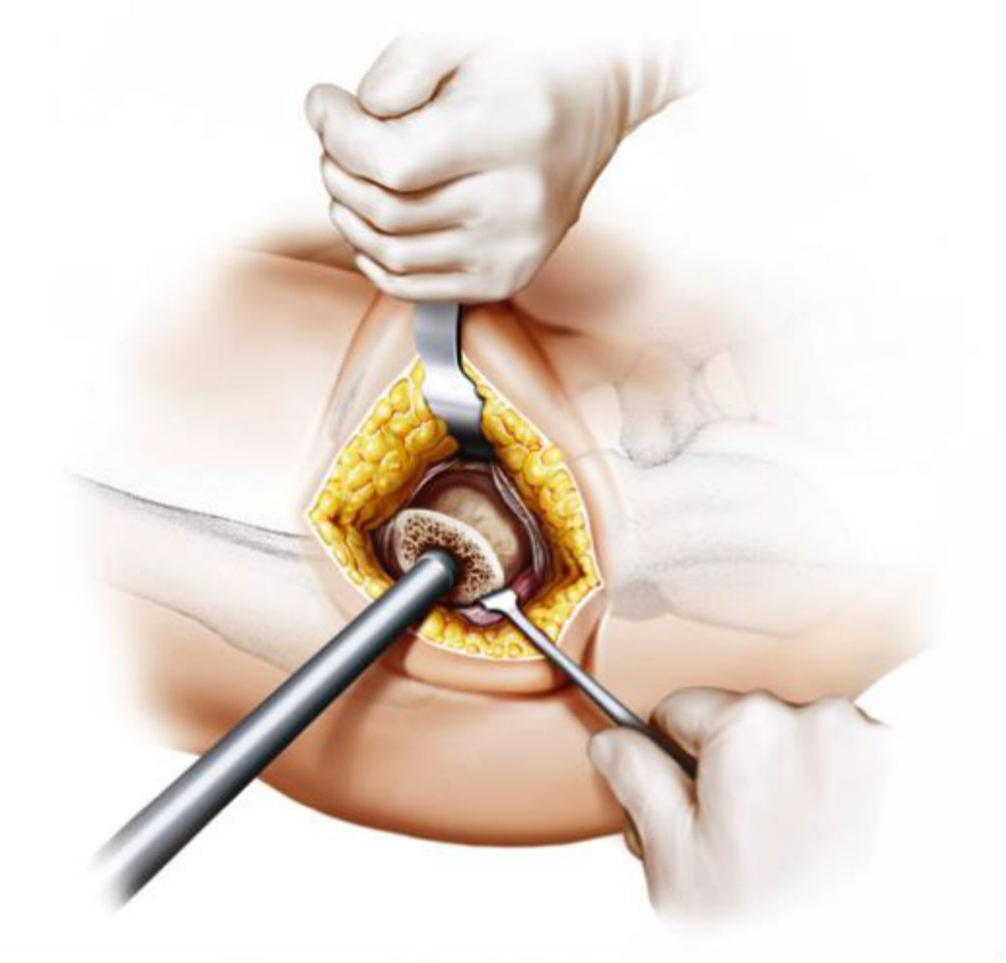
The femoral head is removed from the acetabulum using the femoral head extractor. To protect the gluteus medius a Langenbeck retractor is placed medially and pulled proximally. From [15]

**Fig. 13:**
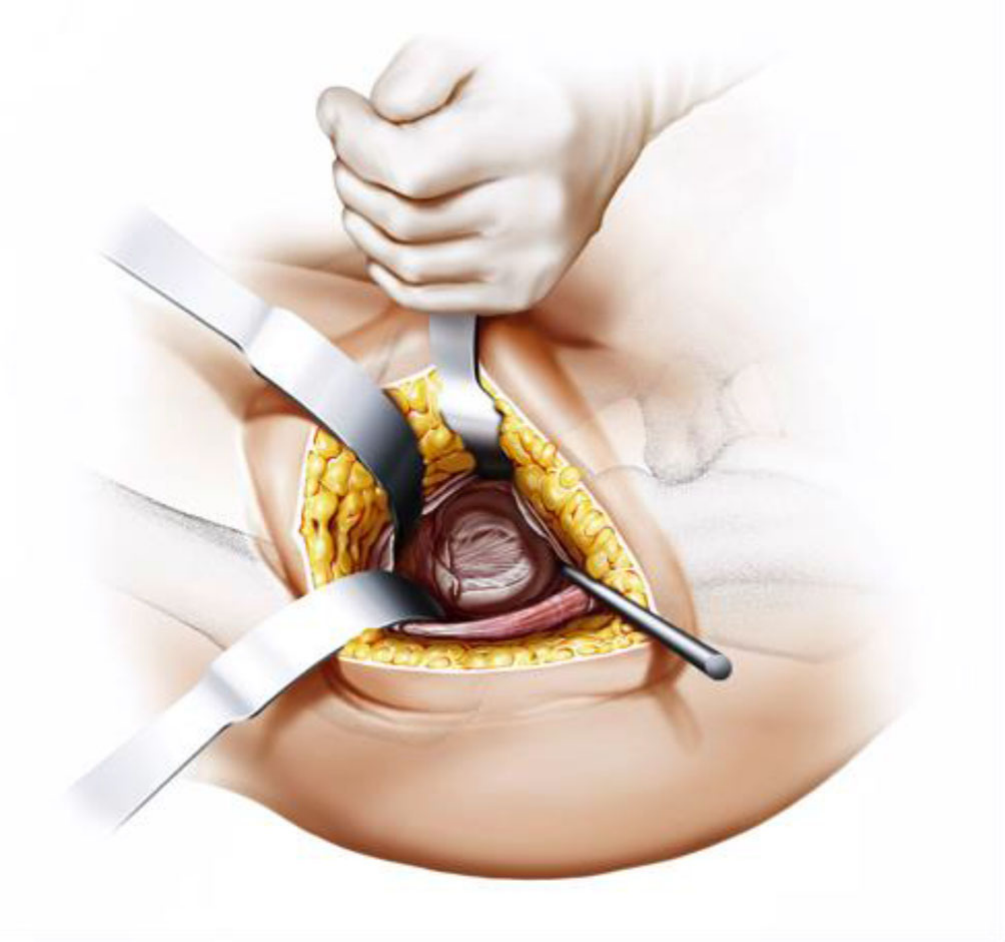
During acetabular preparation a Steinmann pin is inserted in the proximal end of the acetabulum to provide optimal protection to the gluteal muscles. Two curved retractors distally and dorsally support the acetabular exposure. From [15]

**Fig. 14:**
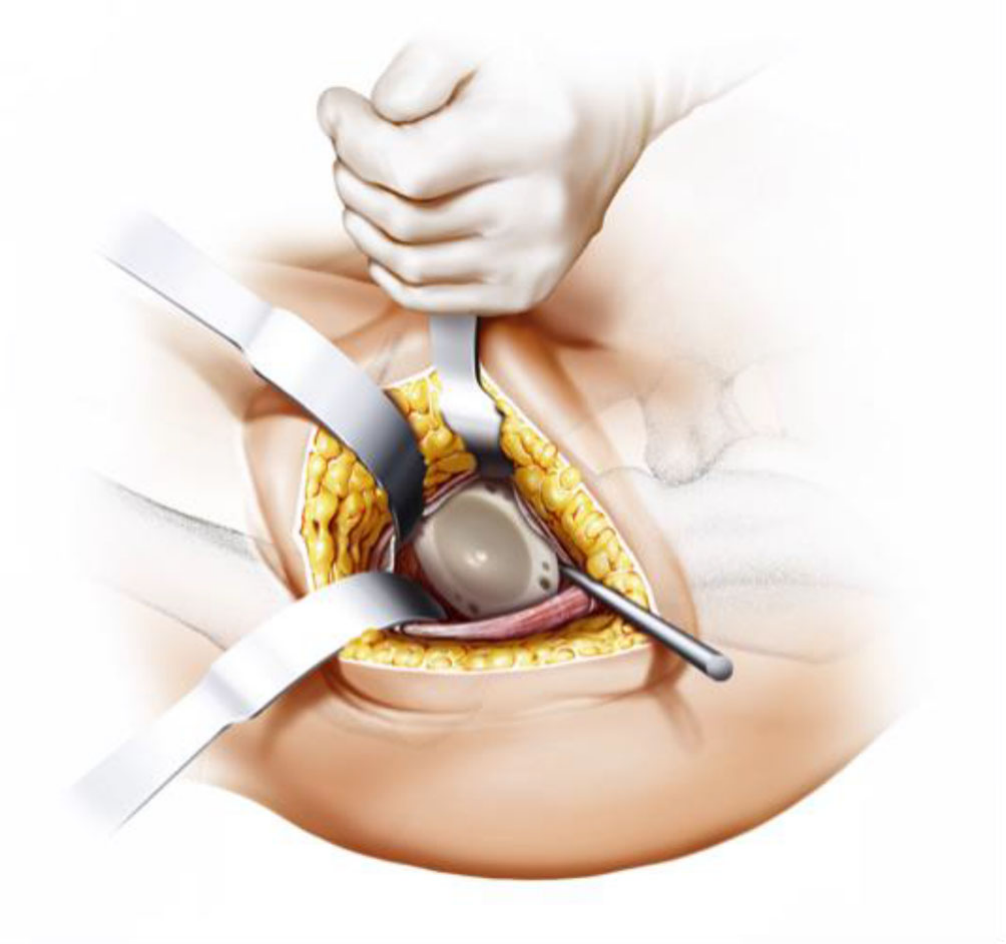
After acetabular preparation the cup is implanted in approximately 45° inclination and 10° anteversion according to the preoperative templating and depending on patient’s individual anatomy. (Courtesy of Mathys Ltd Bettlach)

**Fig. 15:**
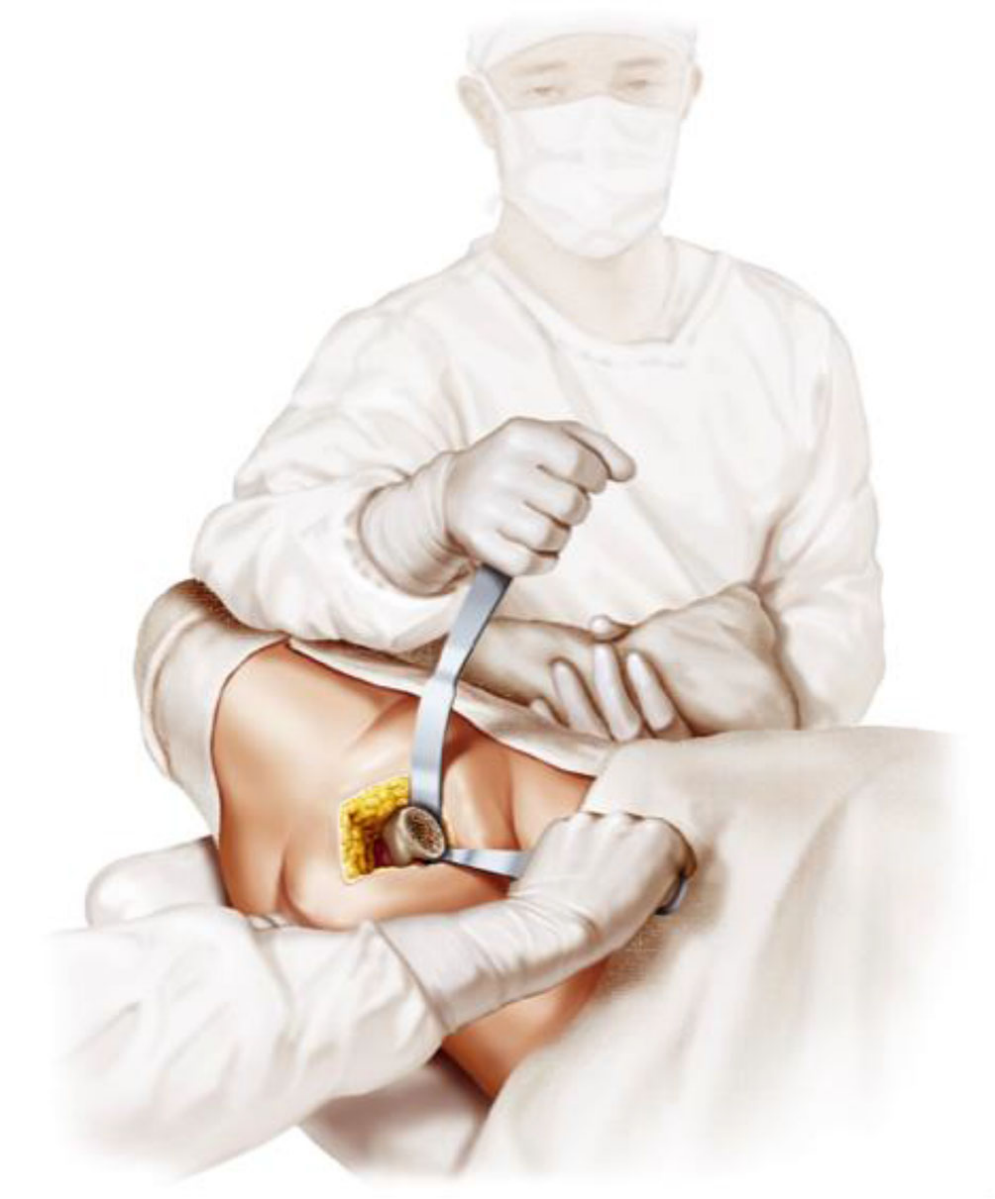
For femoral preparation first the knee roll is removed and the contralateral leg is hyperextended about 15° lowering the leg support. After performing a 90° external rotation and a maximum of 90° flexion of the knee joint, the leg is held in maximum adduction by the second assistant. (Courtesy of Mathys Ltd Bettlach)

**Fig. 16:**
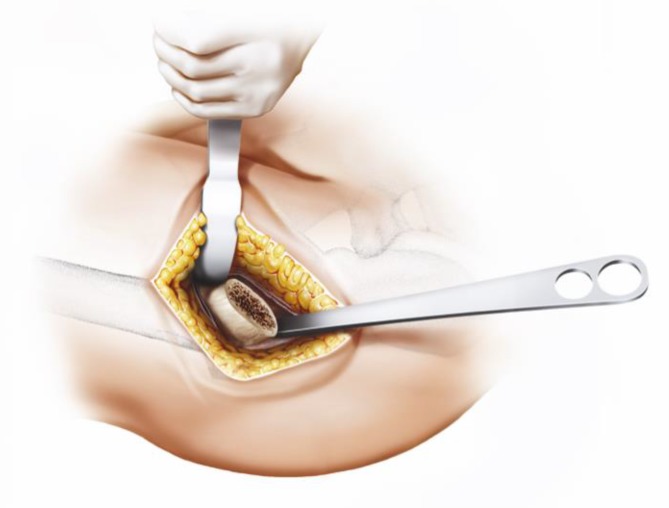
A curved retractor is positioned on the medial side of the proximal femoral neck while a long straight retractor is positioned proximally at the posterior (medial) cortical end of the femoral neck. Note that there is no contact to the greater trochanter at all, distinctly minimizing the risk of possible damage to the bone and the muscle insertions. From [15]

**Fig. 17:**
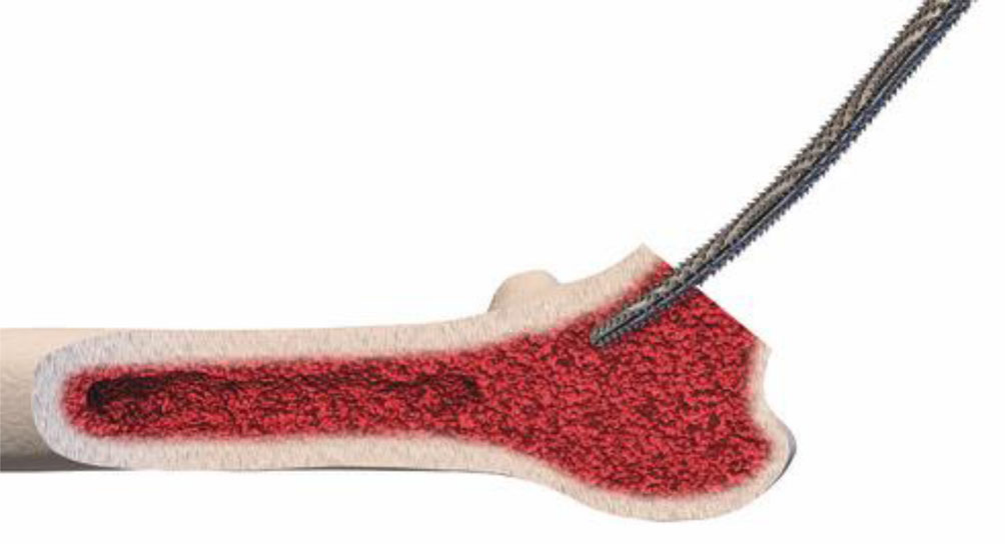
Using the specially curved opening awl, the proximal femur is opened alongside the calcar in the “round-the-corner” technique. The insertion is done anteriorly, not affecting posterior structures such as the greater trochanter or the gluteal muscles. From [15]

**Fig. 18:**
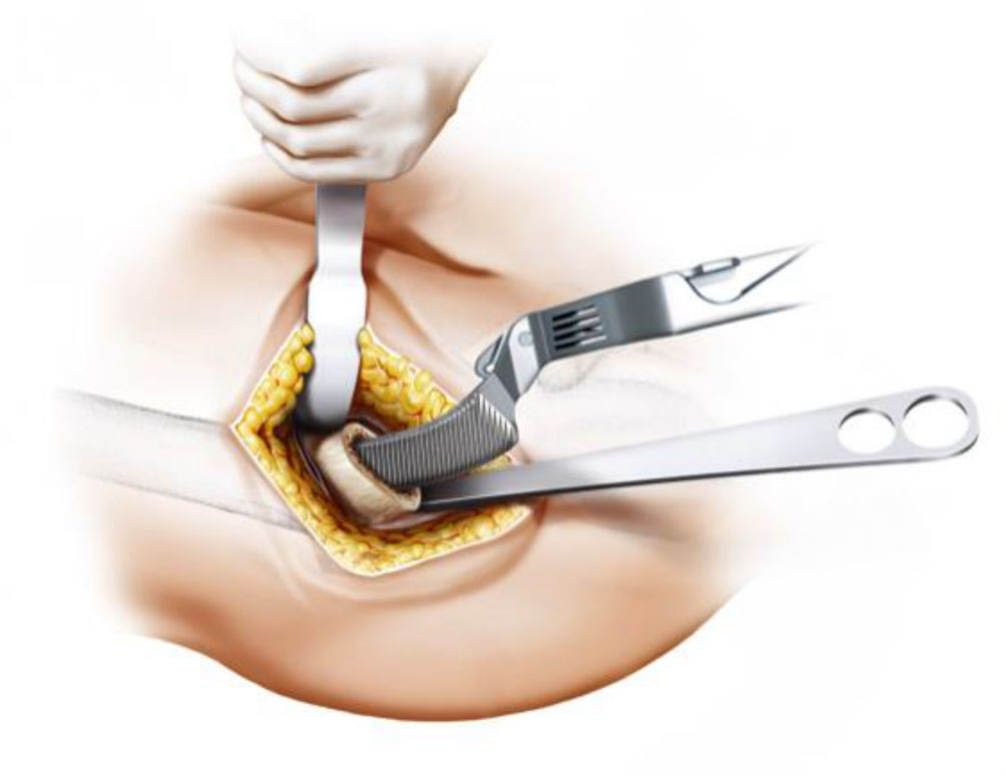
Specially curved, implant-shaped rasps are driven in gently in ascending sizes using a hammer in order to prepare the proximal femur and the femoral canal until cortical contact and a stable fit and fill is reached. A double offset minimally invasive rasp handle is available. From [15]

**Fig. 19:**
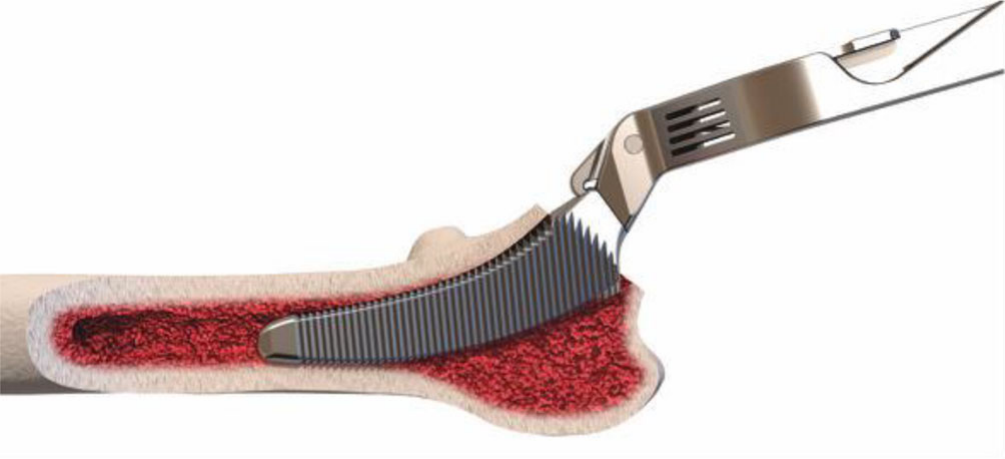
The insertion takes place guided by the calcar in the “round-the-corner” technique. Again, the greater trochanter accompanied by the gluteal muscles is out of reach and therefore can be optimally protected. From [15]

**Fig. 20:**
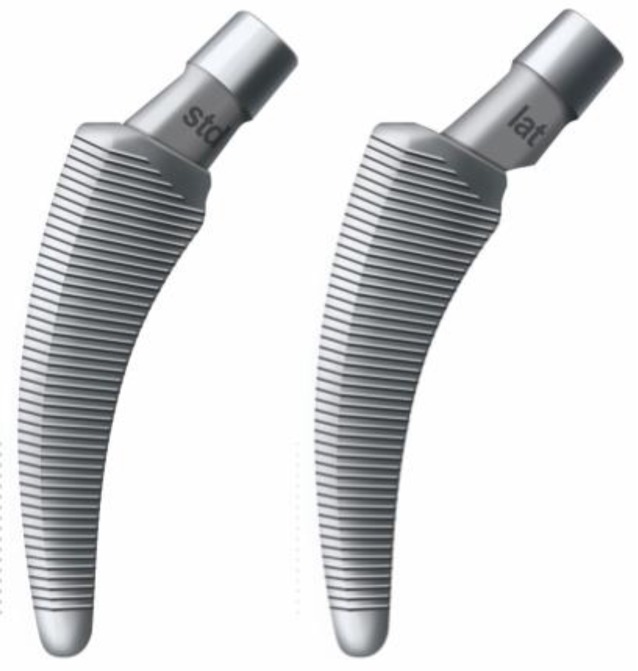
One of two different offset versions (standard and lateral offset) of trial cones can be chosen according to the preoperative templating. (Courtesy of Mathys Ltd Bettlach)

**Fig. 21:**
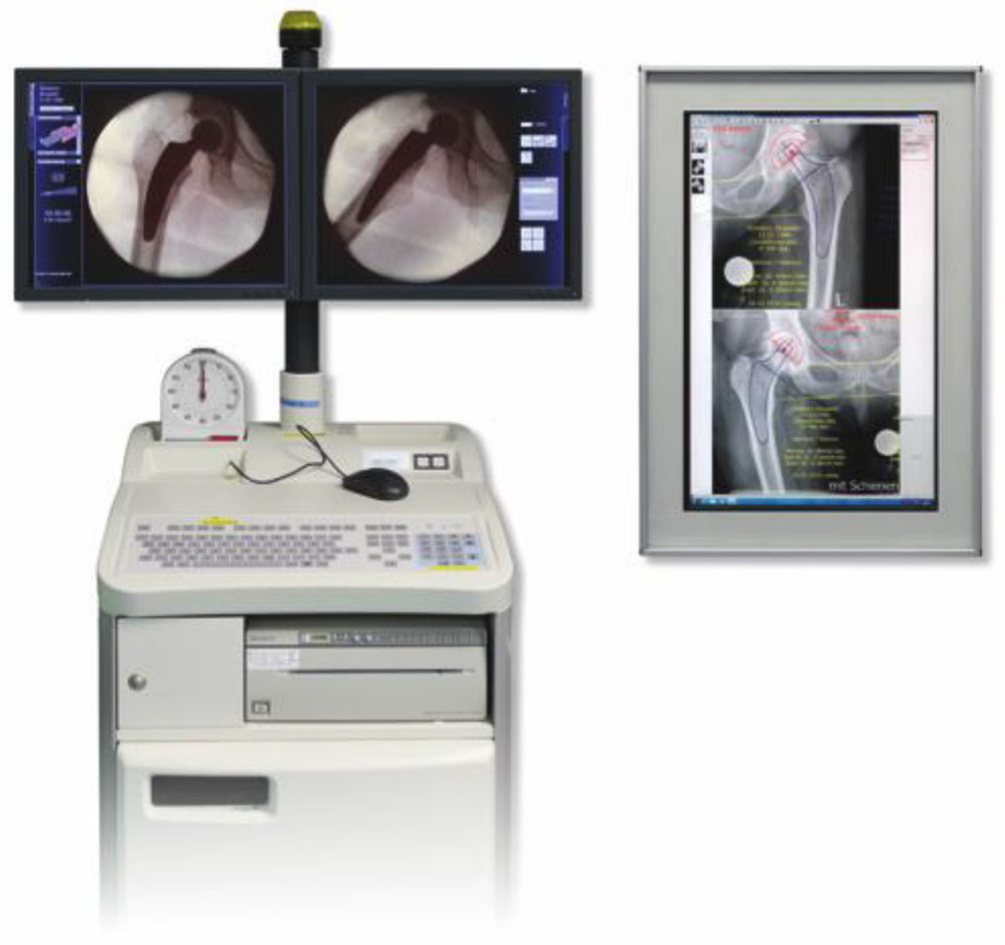
After inserting a head and performing a trial reduction, the assessment of an intraoperative single shot radiograph using a digital image intensifier is highly recommended at this point in order to compare the positioning of the rasp (trial implant) to the preoperative templating. Offset and leg length can be verified and, if necessary, changes can be made. Note that risk of postoperative subsidence might be increased in cases not reaching cortical contact. This should be taken into consideration choosing the correct implant size. (Courtesy of Mathys Ltd Bettlach)

**Fig. 22:**
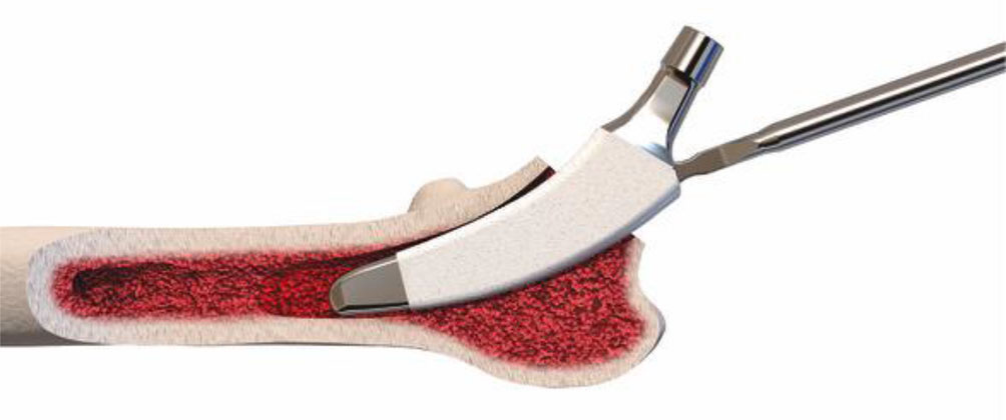
After removing the trial rasp, the definitive implant containing the chosen offset version is inserted anteriorly alongside the calcar using the special implant impactor. From [15]

**Fig. 23:**
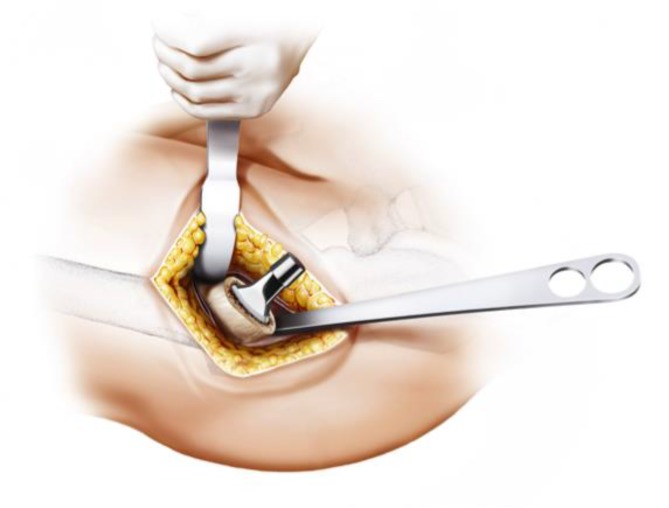
The definitive calcar-guided short-stem positions itself exactly like the trial rasp. After final reduction and wound closure the procedure of the first hip is completed. Upon consultation of the anesthesiologist about the patient’s general condition, surgery can be performed in the same way on the contralateral hip. From [15]

**Fig. 24:**
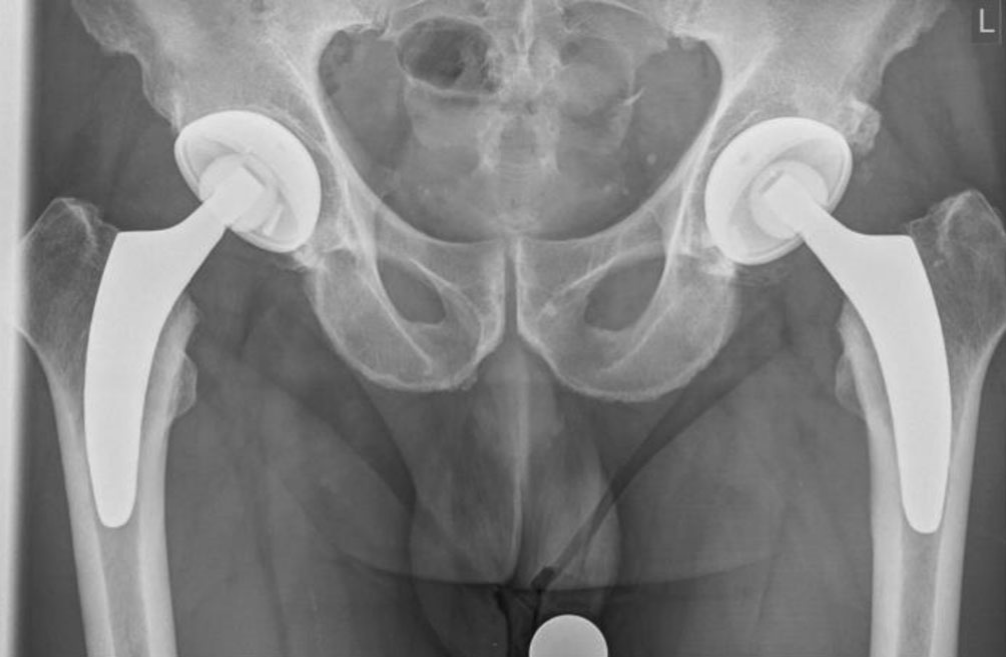
Postoperative radiograph of a one-stage bilateral calcar-guided short-stem THA

## Special surgical considerations

- Glove change should be performed every half hour and before switching position and starting with the second hip in regard to prophylaxis of infection.
- Before the operation the leading symptomatic hip should be identified; it is the one to start off with. In case of complications the procedure of the contralateral hip might not be possible.
- Intraoperative radiograph using digital image intensifier is highly recommended to verify correct trial implant positioning and to assure correct offset and leg length. Therefore intraoperative radiographs are compared to the preoperative templating and possible adjustments can be done (Fig. 21).

## Postoperative management

- Full weight-bearing ambulation allowed under physiotherapy surveillance on day 1, using walker or two crutches
- Functional passive and active motion with initial restriction of flexion of 90°
- Intensive protocol of physiotherapy and rehabilitation
- Wound drainages until postoperative day 1
- Wound dressings until postoperative day 2
- Patients are instructed to climb stairs as soon as possible
- Radiographic assessment after 3–5 days (deep pelvis)
- Average time until discharge: 7 days
- Venous thromboembolic prophylaxis is administered for 28 days
- Skin staples are removed after 14 days
- Radiographic follow-up after 3 days and 6 weeks (deep pelvis)

## Errors, hazards and complications

- Damage to the gluteus superior nerve, which supplies the gluteus medius, gluteus minimus and the tensor fasciae latae muscles.
- After completion of the first side, further processing takes place only upon consultation of the anesthesiologist in order to confirm a stable patient. In case of complications the contralateral hip is not to be operated.
- The implant needs sufficient fit and fill into the femoral bone with tight cortical contact in order to avoid subsidence given immediate full weight bearing.
- Learning curve is necessary in order to avoid malpositioning of the implant.

## Results

**Fig. 25:**
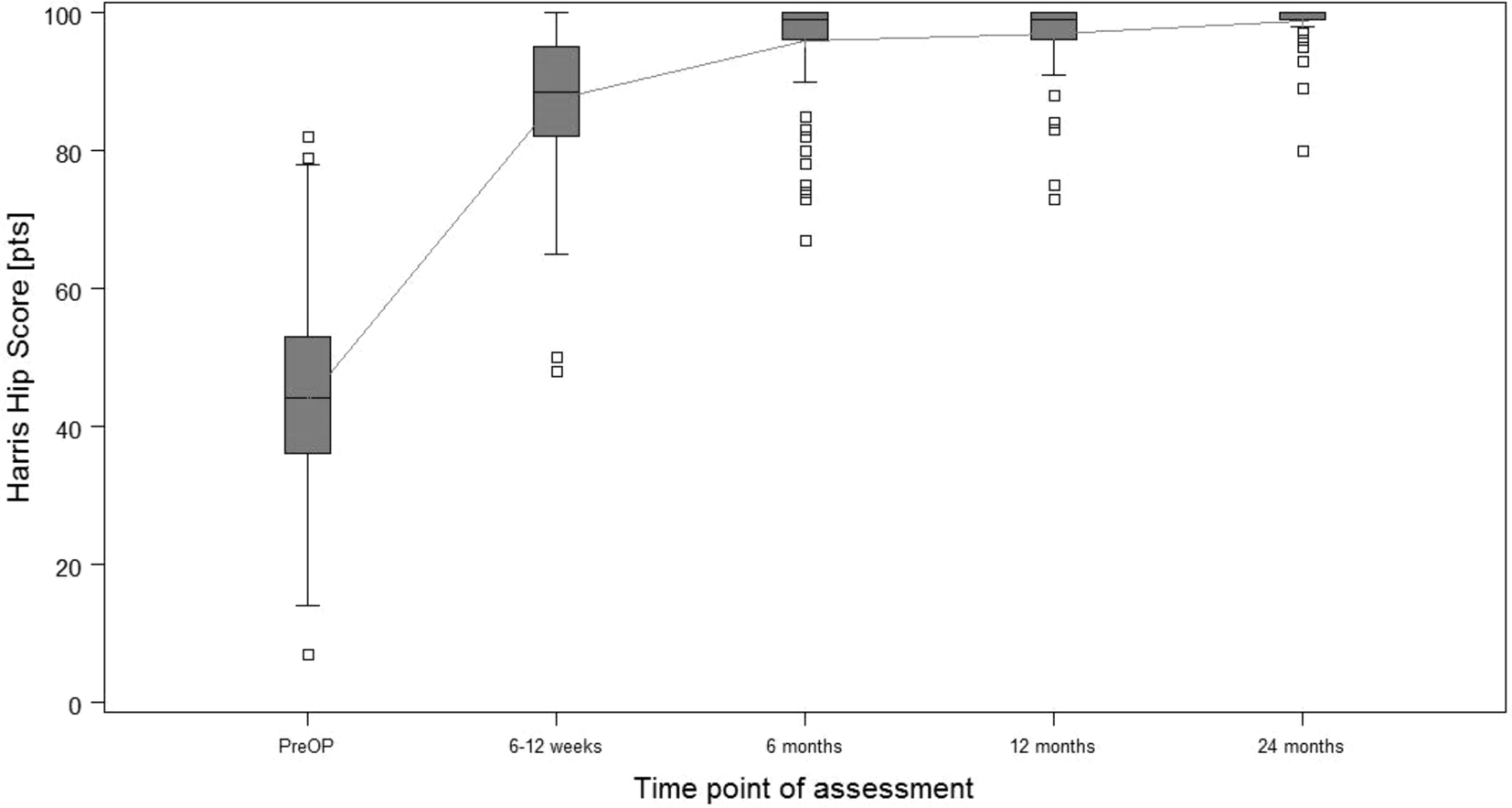
Box plot of the Harris Hip Score over a follow-up of 24 months. *Pts* patients. (Courtesy of Mathys Ltd Bettlach)

**Fig. 26:**
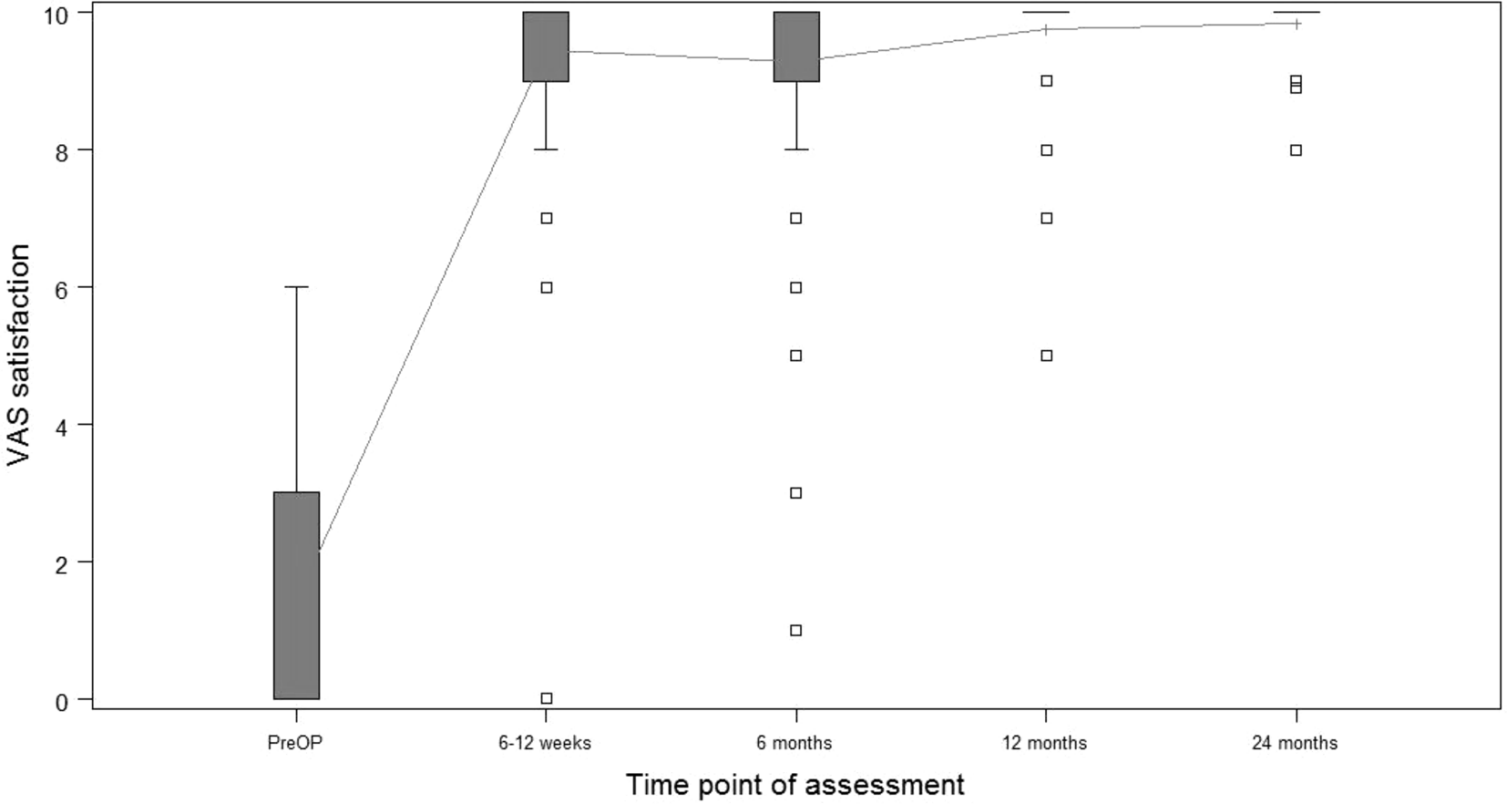
Box plot of satisfaction on the Visual Analogue Scale (*VAS*) over a follow-up of 24 months. (Courtesy of Mathys Ltd Bettlach)

Starting in December 2010 until today the introduced calcar-guided short-stem (optimys, Mathys Ltd., Bettlach, Switzerland) has been implanted using the presented approach and implantation technique in over 4000 cases at the authors’ institution. In almost 500 patients the implantation was done one-stage bilaterally. The first 54 consecutive one-stage bilateral cases (108 hips) were included in a prospective observational study analyzing clinical and radiological results. The mean age at surgery was 62.7 years (standard deviation [SD] 9.0; range 36.7–76.8 years) and the mean operation time was 44.6 min (SD 16.6; range 19.0–96.0 min) for each hip. The 2‑year results (mean 28.5 months), including the learning curve, have been analyzed so far and preliminary results have previously been published [9]. The follow-up was performed after 6 weeks, 6 months, 12 months and 24 months. After 6 weeks mean Harris Hip Score (HHS) and mean satisfaction on visual analogue scale (VAS) were already 87.4 (SD 9.9; range 48.0–100.0) and 9.4 (SD 1.2; range 0–10) respectively (Figs. 25 and 26). Initial clinical function is encouraging, allowing patients to be self-dependent a few days after surgery. After 2 years the values improved further to a mean HHS of 98.8 (SD 3.2; range 80.0–100.0) and mean satisfaction on VAS of 9.9 (SD 0.5; range 8.0–10.0) (Figs. 25 and 26). In the radiological follow-up after 2 years the incidence of typical radiological alterations, like stress-shielding and cortical hypertrophy in total is low, suggesting a physiological load distribution in the proximal femoral bone [11]. An analysis of the potential of reconstructing patient’s anatomy showed that the technique of individualized positioning using a calcar-guided short-stem is able to reconstruct femoro-acetabular offset and leg length in a broad range [10].

The overall complication rate is low. One patient showed an intraoperative avulsion of the greater trochanter on one side, without any clinical malfunction. No therapy was required. One case of deep vein thrombosis (DVT) was reported despite regular medical prophylaxis, which could be treated successfully. In addition a prolonged seroma was documented in three cases. No postoperative joint infection occurred in any of the patients. To date, no revision surgery was needed. No signs of aseptic loosening or any other implant failure was observed. Mean drop of haemoglobin measured 5.3 g/dl. Seven patients (12.9%) needed at least one blood transfusion. However, in those patients included in the study in 2010, the usage of tranexamic acid had not been implemented yet. Thus, rates of haemoglobin drop and blood transfusion are possibly reduced further nowadays.

The muscle-sparing combination of the MIS approach and the special “round-the-corner” technique using the calcar-guided short-stem in one-stage bilateral procedure leads to extraordinary fast mobilization and rehabilitation with excellent early clinical results and distinctly high satisfaction rates. After overcoming the learning curve the implantation of a calcar-guided short-stem using the MIS anterolateral approach presents as an easy and fast technique with a low intraoperative complication rate, compared to non-curved straight-stem designs, which is favorable in regard to the usage in one-stage bilateral procedures. Mid- and long-term results are still awaited; therefore further follow-up is mandatory and will be continued.
